# Cytotoxicity and effect on wound re‐epithelialization after topical administration of tranexamic acid

**DOI:** 10.1002/bjs5.50192

**Published:** 2019-09-26

**Authors:** T. A. Eikebrokk, B. S. Vassmyr, K. Ausen, C. Gravastrand, O. Spigset, B. Pukstad

**Affiliations:** ^1^ Faculty of Medicine Norwegian University of Science and Technology Trondheim Norway; ^2^ Department of Circulation and Medical Imaging Norwegian University of Science and Technology Trondheim Norway; ^3^ Department of Clinical and Molecular Medicine Norwegian University of Science and Technology Trondheim Norway; ^4^ Section for Plastic and Reconstructive Surgery, Clinic of Surgery St Olav's University Hospital Trondheim Norway; ^5^ Department of Clinical Pharmacology St Olav's University Hospital Trondheim Norway; ^6^ Department of Dermatology St Olav's University Hospital Trondheim Norway

## Abstract

**Background:**

Topical administration of tranexamic acid (TXA) reduces bleeding from surgical wounds similarly to intravenous use, but with negligible risk of adverse systemic events. Topical use is expanding, but is off‐label. Surgeons lack guidelines regarding safe topical dosages and modes of administration. The effects of topical TXA on skin cells and wound healing are unknown. This study investigated whether topical TXA might be cytotoxic or affect wound re‐epithelialization.

**Methods:**

Human keratinocytes and fibroblast cell cultures and an *ex vivo* human skin wound model were subjected to both short (limited) and long (chronic) exposure to various clinically relevant concentrations of TXA to mimic different modalities of topical administration. Cytotoxicity and effects on wound re‐epithelialization were evaluated.

**Results:**

In cell culture, toxicity from chronic exposure was associated with increasing concentration and exposure time. Limited exposure to TXA did not cause significant cytotoxicity even at high concentrations. Re‐epithelialization was completely absent in wounds chronically exposed to TXA concentrations of 25 mg/ml or above, and 50–100 mg/ml induced epidermolysis of normal epithelium, possibly by a non‐toxic mechanism. Wound re‐epithelialization was slightly delayed, but not impaired, by limited exposure to 100 mg/ml or chronic exposure to 6·25 mg/ml.

**Conclusion:**

Although short exposure to even high concentrations of topical TXA seems well tolerated *in vitro*, prolonged exposure can be cytotoxic and may affect wound re‐epithelialization. Surgeons should adjust the TXA concentration to the planned mode of topical administration in clinical practice.

**Surgical relevance**

Topical tranexamic acid (TXA) may reduce bleeding from surgical wounds similarly to intravenous administration without the risk of systemic effects. Little is known, however, regarding the adverse effects of TXA on exposed tissues.

We exposed *in vitro* human keratinocytes and fibroblasts and an *ex vivo* human skin wound model to TXA at various concentrations and time intervals and found that short exposure to even high concentrations or prolonged exposure to low concentrations of TXA was well tolerated. Prolonged exposure to increasing concentrations increased keratinocyte and fibroblast toxicity, and TXA concentrations of 25 mg/ml or above completely prevented wound‐re‐epithelialization.

Prolonged exposure to high concentrations of topical TXA may exert unwanted local tissue effects. This study suggests that surgeons should adjust TXA concentration to the planned mode of topical administration in clinical practice.

## Graphical abstract

Topical tranexamic acid reduces bleeding from surgical wounds, but little is known about possible adverse effects on the exposed tissue. In this experimental study, prolonged *in vitro* exposure was cytotoxic to keratinocytes and fibroblasts, and prevented wound re‐epithelialization in a dose‐dependent manner, whereas short exposure was well tolerated even at high concentrations.

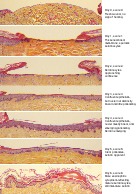

Correct concentration of topical tranexamic acid is the key

## Abstract (es)

**Antecedentes:**

La administración tópica de ácido tranexámico (*tranexamic acid*, TXA) reduce la hemorragia de las heridas quirúrgicas de forma equivalente a su uso endovenoso, pero con un riesgo insignificante de eventos adversos sistémicos. El uso tópico se está expandiendo, pero se realiza fuera de indicación. Los cirujanos no disponen de directrices sobre las dosis para uso tópico seguras y las formas de administración. Se desconocen los efectos del TXA tópico sobre las células de la piel y sobre la curación de las heridas. Nos propusimos investigar si el TXA tópico puede ser citotóxico o afectar la reepitelización de la herida.

**Métodos:**

Los cultivos de queratinocitos humanos y fibroblastos y un modelo ex vivo humano de herida en la piel se sometieron a una exposición corta (limitada) y larga (crónica) de varias concentraciones clínicamente relevantes de TXA para simular diferentes modalidades de administración tópica. Se evaluaron la citotoxicidad y los efectos sobre la reepitelización de la herida.

**Resultados:**

En los cultivos celulares, la toxicidad de la exposición crónica se correlacionó con el incremento de la concentración y el tiempo de exposición. La exposición limitada al TXA no causó toxicidad significativa incluso a elevadas concentraciones. No se observó reepitelización en heridas expuestas de forma crónica a concentraciones de TXA de 25 mg/ml o superiores, y 50‐100 mg/ml provocó epidermólisis del epitelio normal, posiblemente por un mecanismo no tóxico. La reepitelización de la herida se retrasó ligeramente, pero no se deterioró por una exposición limitada de 100 mg/ml o exposición crónica de 6,25 mg/ml.

**Conclusión:**

Mientras que la exposición corta, incluso hasta concentraciones elevadas, de TXA tópico parecen ser bien toleradas in vitro, la exposición prolongada al TXA tópico puede ser citotóxica y afectar la reepitelización de la herida. Los cirujanos deben ajustar la concentración de TXA al modo previsto de administración tópica en la práctica clínica.

## Introduction

Tranexamic acid (TXA) has been available as an antifibrinolytic drug for more than 50 years, and its use has attracted greatly renewed interest in the past decade^1^. TXA binds to plasminogen and prevents its conversion to plasmin, thus inhibiting fibrinolysis. Intravenous use to reduce bleeding is particularly common in high‐risk procedures such as joint replacement surgery and cardiothoracic surgery, reducing both bleeding and the need for blood transfusions by about one‐third^2^.

Fear of possible adverse effects may lead to reluctance to use intravenous TXA routinely in all surgical procedures, although no increased risk of thromboembolic events has been demonstrated in large randomized studies and meta‐analyses^2^, ^3^, ^4^. TXA can, however, cross the blood–brain barrier, and a dose‐dependent risk of non‐ischaemic seizures has been reported^5^, ^6^. Topical contact with the central nervous system should be discouraged, as both accidental intrathecal administration of TXA in humans^7^ and subdural use of fibrin sealants containing TXA in animal studies^8^ have caused epileptic convulsions.

Topical administration of TXA provides a high concentration at the application site where the antifibrinolytic effect is needed, but a low systemic concentration with an assumed reduced risk of systemic side‐effects^9^. Studies on topical use have been published, particularly from orthopaedic and cardiothoracic surgery, and experience of use in other surgical specialties is increasingly emerging^10^. Although reviews from joint replacement surgery suggest that the route of TXA administration is immaterial with regard to both efficacy and safety^11^, ^12^, further investigation is required on topical use in terms of dosage regimens and risk of adverse effects^13^, ^14^. Surgeons have few guidelines, as topical use is still off‐label.

The recommended prophylactic dose of intravenous TXA in surgery is 10–20 mg/kg^15^, which gives a peak plasma concentration of 80–160 μg/ml^16^. Some studies in cardiac surgery have advocated maintaining plasma levels above 150 μg/ml^17^. Even with maximum intravenous dosing, plasma concentrations will rarely exceed 200 μg/ml. When administrating TXA topically, existing practice is to dilute vials for intravenous use (100 mg/ml) to concentrations of 5–50 mg/ml^18^, thereby applying tenfold to 100‐fold higher concentrations of TXA on to the wound surface. The lowest TXA concentration needed for a topical haemostatic effect is unknown, but 1–5 mg/ml has been shown to have an effect^18^.

Few studies have explored the potential local toxicity of topical TXA. Chondrocyte toxicity has been investigated^13^, ^19^, ^20^, as topical use of TXA is expanding in orthopaedic arthroplasty surgery. Chondrocyte toxicity increases with both concentration and exposure time, and 20–25 mg/ml may represent a threshold value^13^, ^20^. As topical TXA may reduce bleeding from many types of wound, research is needed on other cell types.

The effect of topical TXA on cells essential for wound healing has not yet been explored. The aim of this study was to investigate whether TXA, applied directly to primary cells derived from human skin and to a full‐thickness human skin wound model, has the potential to be cytotoxic and/or may affect wound re‐epithelialization. A secondary objective was to determine a potentially safe concentration for topical use if cytotoxicity was identified.

## Methods

Modes of topical administration of TXA include instilling a bolus into a closed wound cavity or irrigating the wound for a particular length of time^18^. The authors have previously published a moistening method in which TXA 25 mg/ml is smeared on to a wound surface to leave a thin film of drug directly before closure^21^. The topically applied concentration will be diluted by local tissue fluids and the drug will be absorbed gradually. A moistening film will be diluted and absorbed rapidly, representing a limited exposure, whereas a bolus administration into a closed wound cavity will be diluted more slowly and may remain in contact with the wound surface for much longer, representing a chronic exposure^9^.

In this study, cell cultures and a human skin wound model were exposed to TXA in two different ways to mimic different modes of topical administration. For chronic exposure, wounds and cells were subjected to a growth medium containing TXA, mimicking a topical bolus administration. For limited exposure, wounds and cells were exposed to TXA for 10 min, after which the TXA‐containing solution was discarded and TXA‐free growth medium was added, mimicking a moistening administration.

### Cell models

*Appendix* *S1* (supporting information) provides full details of cell culture viability and cytotoxicity assays.

Commercially available human keratinocytes and fibroblasts were investigated *in vitro* using methyl thiazolyl tetrazolium (MTT) and lactate dehydrogenase (LDH) assays. MTT is converted to an insoluble purple formazan in the mitochondria of healthy cells. The amount of formazan is proportional to cell metabolism and indirectly to viability. Loss of LDH through the cell membrane indicates cell damage. The concentration of LDH is used to measure cytotoxicity.

#### *Chronic exposure to tranexamic acid*

In clinical practice, standard vials of TXA for intravenous use (100 mg/ml dissolved in sterile water) are diluted in 0·9 per cent sodium chloride to the desired concentration and volume. Prolonged exposure to a solution with no nutrients will starve the cells *in vitro*. The solution for chronic exposure was therefore made by diluting powdered TXA (Toronto Research Chemicals, North York, Ontario, Canada) in growth medium to a concentration of 100 mg/ml. Further dilution with pure medium in a twofold series yielded final TXA concentrations of 100, 50, 25, 12·5 and 6·25 mg/ml. Some 150 μl TXA medium was added to the wells. Pure medium was used as control in all series.

#### *Limited exposure to tranexamic acid*

Vials containing TXA 100 mg/ml (Stragen, Hillerød, Denmark) were diluted with 0·9 per cent sodium chloride in a twofold series, yielding final TXA concentrations of 100, 50, 25, 12·5 and 6·25 mg/ml. Some 150 μl was added to each well. After 10 min the TXA solution was discarded. As the wells were not rinsed, a fluid film containing TXA (measuring on average 9 μl) remained. Fresh growth medium was added, and the cells were thus exposed to an estimated 6–10 per cent of the initial concentration for the remaining observation period.

To differentiate between the effect of TXA and that of the sterile water in standard TXA vials or the dilution by 0·9 per cent sodium chloride, limited exposure series were also performed exposing the cells to equal concentrations of powdered TXA dissolved in medium. The control for all series was exposure to growth medium, with the same fluid exchange procedure using growth medium only.

For all cell studies, viability and cytotoxicity were evaluated after 24, 48 and 72 h. Each concentration was studied in five parallel wells, and the studies were repeated in three separate series.

### *Ex vivo* skin wound model

To evaluate cytotoxicity in a complex model more similar to the *in vivo* situation, a novel *ex vivo* human skin model adapted from that of Jansson and colleagues^22^ was used. Human skin donated from patients undergoing skin‐reducing surgery was used to make superficial wounds in full‐thickness skin. This part of the study was approved by the Regional Committee for Medical and Health Research Ethics (2009/1210/REKmidt), and informed consent was obtained from all donors.

**Figure 1:**
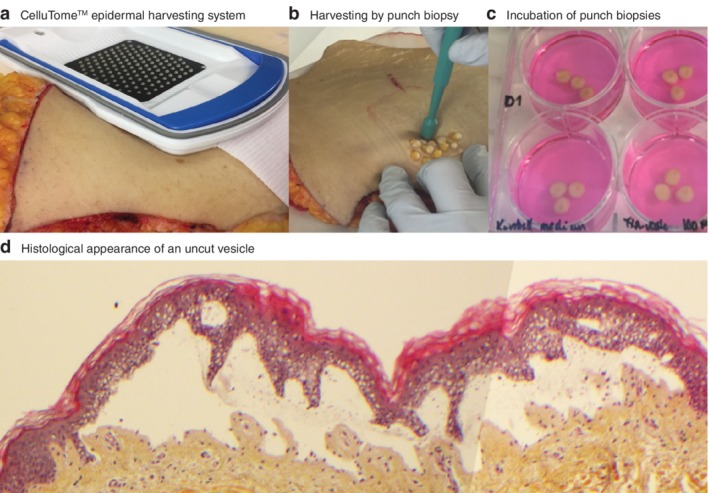
The human skin wound model **a** The CelluTome™ epidermal harvesting system attached to skin donated from an abdominoplasty; superficial vesicles emerge from vacuum suction and are cut by an incorporated tangential knife for epidermal harvesting for skin grafting. **b** The resulting superficial wounds are harvested by punch biopsy. **c** Punch biopsies with a centralized wound are incubated in medium at 37°C. **d** Histological appearance of an uncut vesicle, demonstrating the vacuum‐induced epidermal release along the basal membrane (haematoxylin and eosin staining; magnification ×10).

To standardize the depth and size of the wounds, the CelluTome™ (Kinetic Concepts, San Antonio, Texas, USA) epidermal harvesting system was used. This system creates epidermolysis through vacuum suction and thus produces superficial wounds with an intact basal membrane. Each wound with surrounding skin was cut out with a 6‐mm punch biopsy (*Fig*. 1). Biopsies were placed in wells containing 3 ml treatment or control medium, and the wounds were left to re‐epithelialize in a 37°C incubator. Each concentration was studied in three biopsies embedded in the same well, and the studies were repeated in three separate series, with skin from three different donors. The wounds were harvested after 1, 4 and 8 days. The biopsies were then preserved in formalin for haematoxylin and eosin (HES) staining, and histological evaluation of re‐epithelialization. Owing to unexpected findings, complementary *post‐hoc* periodic acid–Schiff and cytokeratin (CK) AE1/AE3 staining was performed in selected wounds. Details of staining protocols are provided in *Appendix* *S2* (supporting information).

**Figure 2:**
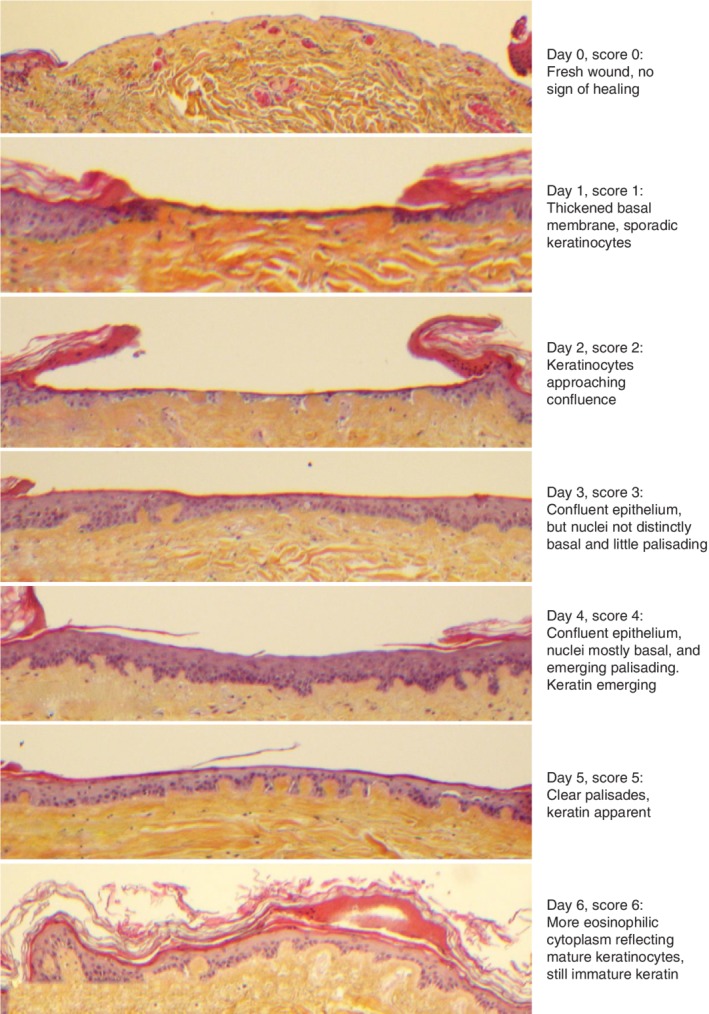
Scoring system for the human skin wound model Histological appearance of healing observed over time in control wounds exposed to growth medium only, and a proposed scoring system for evaluating observed healing (haematoxylin and eosin staining; magnification ×10).

A healing score reference sheet was designed, based on the histological characteristics of the re‐epithelialization on different days in biopsies exposed to growth medium only (*Fig*. 2). The score reference sheet is a compilation of biopsies from different series and thus from different donors. All study biopsies were assigned random numbers, and the healing score was evaluated by a person blinded to the randomization. Biopsies without an identifiable wound and biopsies in which a jagged wound surface without basal membrane indicated a deeper wound were excluded from the final analysis.

#### *Chronic exposure to tranexamic acid*

Growth medium containing TXA 100 mg/ml was diluted in medium to final TXA concentrations of 100, 25 and 6·25 mg/ml, and the biopsies were exposed to the medium for the entire observation period. In pilot investigations, concentrations of 50, 10, 4 and 0·8 mg/ml were evaluated in a single series.

#### *Limited exposure to tranexamic acid*

Biopsies were exposed for 10 min to 3 ml of a standard TXA 100 mg/ml vial with exposure to 0·9 per cent sodium chloride as control. After 10 min, the TXA solution was discarded, but a remnant drug film of 20–30 μl remained. Some 3 ml of growth medium was added to the wells, and the biopsies were therefore exposed to an estimated TXA concentration of 6–10 mg/ml for the remaining observation period. Limited exposure was not performed with concentrations lower than 100 mg/ml as pilot studies had indicated little difference from control at this concentration.

### Statistical analysis

For the skin wound studies, differences in healing scores between biopsies exposed to TXA and control biopsies were analysed using the Wilcoxon signed rank test; descriptive data are presented as mean (range). *P* < 0·050 was considered statistically significant. For the cell studies, the difference in cytotoxicity and viability between each TXA concentration and its corresponding control value was calculated and analysed using a linear mixed model. Potentially significant differences in mean values within the separate series were considered by using series as random effects in the linear mixed model. Descriptive data are presented as mean(s.d.) values. Any difference of less than 10 per cent in cytotoxicity or viability between a TXA concentration and control was considered clinically insignificant, even if statistically significant, and was therefore not indicated as significant. All analyses were performed using SPSS® version 25 for Windows® (IBM, Armonk, New York, USA).

## Results

### Cell cultures

#### *Chronic exposure to tranexamic acid*

**Figure 3:**
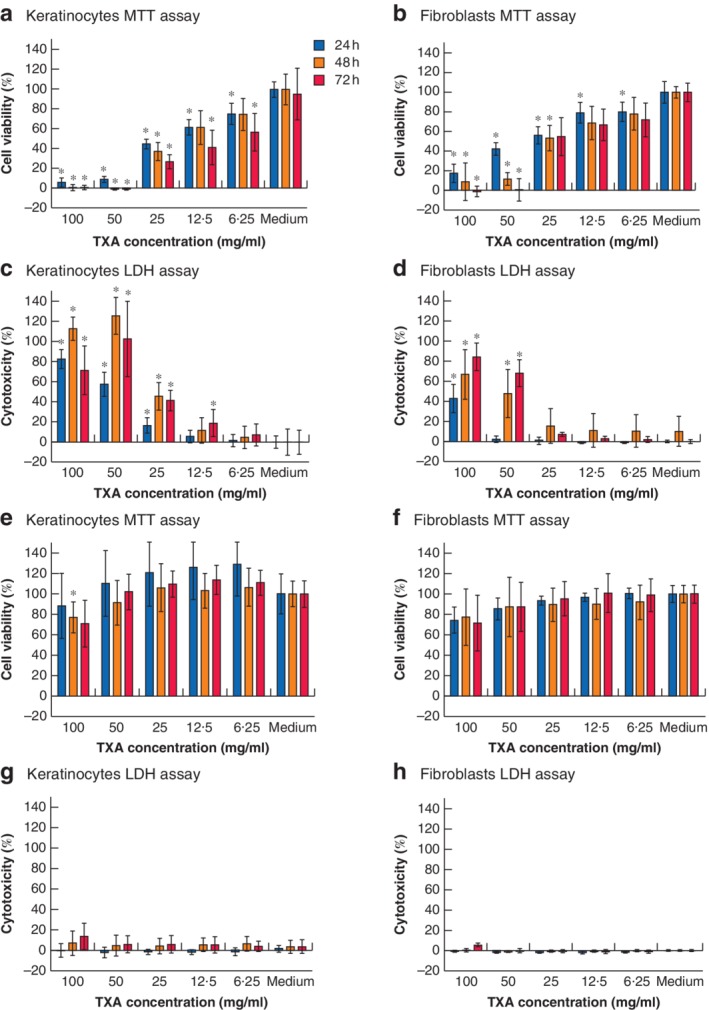
Human epidermal keratinocyte and human dermal fibroblast viability and cytotoxicity assays after chronic and limited exposure to tranexamic acid Viability was assessed by methyl thiazolyl tetrazolium (MTT) assay and cytotoxicity by lactate dehydrogenase (LDH) assay after **a–d** chronic and **e–h** limited exposure to tranexamic acid (TXA). For viability, values are given as percentages of the medium control after 24, 48 and 72 h. For cytotoxicity, values are given as percentages compared with control after 24, 48 and 72 h. Each concentration was studied in five parallel wells, and studies were repeated in three separate series (*n* = 15). *P < 0.05 *versus* corresponding medium control for each group (linear mixed model). Results that are statistically significant versus control but have a less than 10 per cent difference are not marked as they are considered clinically insignificant (*Table* *S2*, supporting information).

Viability and cytotoxicity after chronic TXA exposure is shown in *Fig*. 3 *a–d* and *Table* *S1* (supporting information).

Cell survival decreased with increasing TXA concentration and length of exposure. No cells survived 72 h exposure to TXA 50 mg/ml or above, whereas around 50 per cent of cells survived prolonged exposure to TXA 25 mg/ml. TXA 12·5 mg/ml or below exerted little toxicity on the cells. Fibroblasts were somewhat more robust than keratinocytes.

#### *Limited exposure to tranexamic acid*

Viability and cytotoxicity after limited TXA exposure is shown in *Fig*. 3 *e–h* and *Table* *S2* (supporting information). There was a non‐significant trend towards less viability and increased cytotoxicity after limited exposure to 100 mg/ml, but no effect on either cell type at lower concentrations.

No difference was seen between limited exposure to vial TXA *versus* medium TXA, and the cytotoxic effect of the sterile water component in vials or sodium chloride for dilution was therefore not significant. Results from limited exposure to TXA‐containing medium are presented in *Table* *S3* (supporting information).

### *Ex vivo* skin models

**Figure 4:**
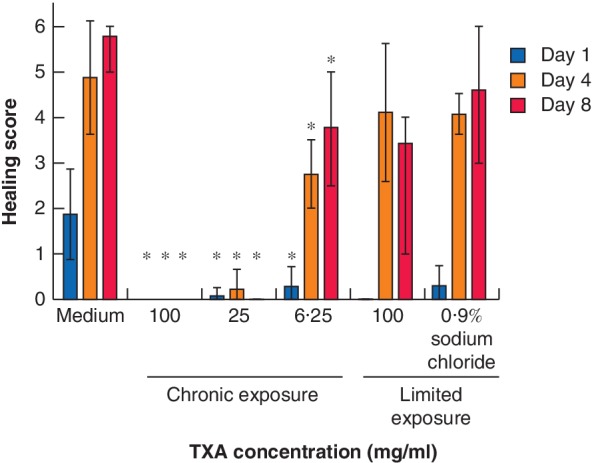
Healing score in *ex vivo* human skin wound biopsies after chronic and limited exposure to tranexamic acid Mean (range) scores were determined according to the scoring system presented in *Fig*. 2. TXA, tranexamic acid. *P < 0·050 *versus* the respective control in each group (Wilcoxon signed rank test).

**Figure 5:**
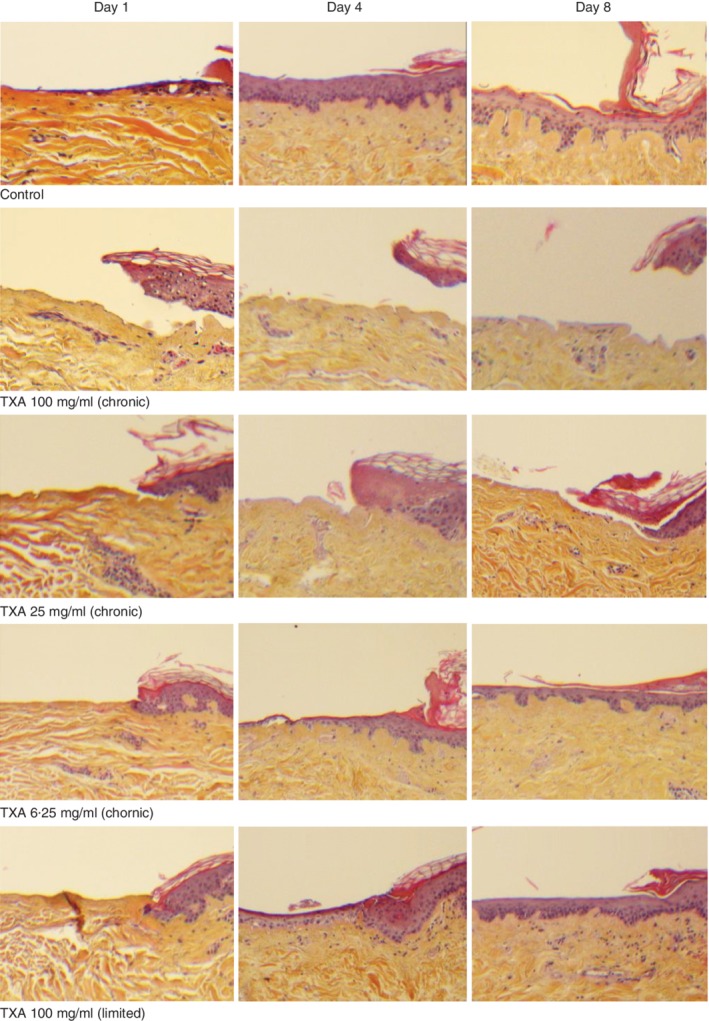
Histological appearance of wound re‐epithelialization after exposure to different concentrations of tranexamic acid in the human wound skin model Wound edge with normal epithelium to the right demonstrating healing on days 1, 4 and 8 after chronic exposure to tranexamic acid (TXA) 100, 25 and 6·25 mg/ml, and after limited exposure to TXA 100 mg/ml (haematoxylin and eosin staining; magnification ×10).

Representative wounds for evaluation were identified in 140 of the 162 biopsies (86·4 per cent). Healing scores for the various TXA concentrations are shown in *Fig*. 4 and *Table* *S4* (supporting information). A visual presentation of the re‐epithelialization at different concentrations is presented in *Fig* 5. There was complete re‐epithelialization after limited exposure to TXA 100 mg/ml. Healing time was no slower than after limited exposure to 0·9 per cent sodium chloride. Complete re‐epithelialization also took place during chronic exposure to 6·25 mg/ml, but with a significantly lower healing score, suggesting a possible delay in healing. In pilot studies, no difference between control and chronic exposure to TXA 0·8, 4·0 or 10 mg/ml was observed, but these were results from only one series with three samples for each concentration.

**Figure 6:**
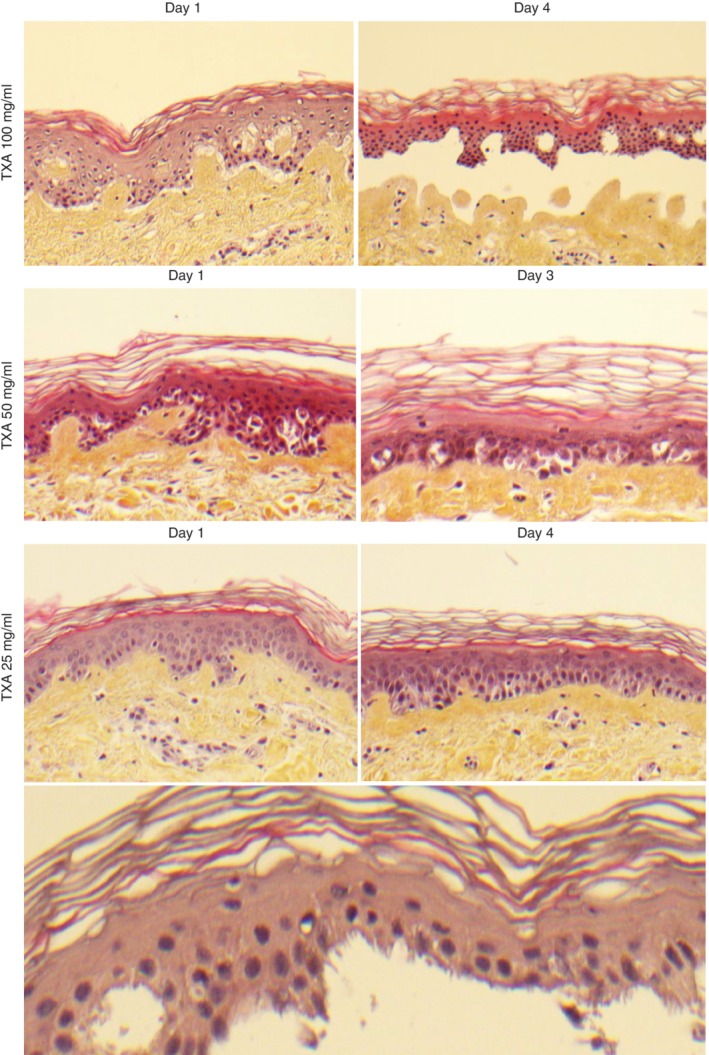
Histological appearance of epitheliolysis in the human skin wound model following chronic exposure to tranexamic acid Normal epithelium not present adjacent to the wound area, demonstrating release and detachment of the epithelial layer along the entire basal membrane by day 4 from chronic exposure to tranexamic acid (TXA) 100 mg/ml, and to a lesser extent from chronic exposure to TXA 50 mg/ml. No clear detachment is seen at day 4 from exposure to TXA 25 mg/ml, 100 mg/ml at day 8 (not shown) was identical to day 3 with complete detachment, intact nuclei and no signs of necrosis. As 50 mg/ml was investigated only in a pilot study, results from day 8 are lacking for this concentration. TXA 25 mg/ml at day 8 (not shown) did not demonstrate consistent signs of detachment compared to control specimens. The bottom image shows the jagged basal surface of detached epithelium (haematoxylin and eosin staining; magnification ×10, except bottom image ×40).

Wound re‐epithelialization did not take place at any time in biopsies chronically exposed to 25 mg/ml or above. Chronic exposure to 100 mg/ml demonstrated emerging epidermolysis along the basal membrane by day 1, and complete epidermolysis and detachment of the epithelium had occurred by day 4. Emerging epidermiolysis was apparent in the pilot 50 mg/ml series by day 3; unfortunately the pilot series did not investigate this dosage beyond day 3 (*Fig*. 6). In the 25 mg/ml series, no definite signs of detachment were seen compared to controls by day 8. HES staining did not suggest cell death in detached epithelium or underlying dermis, and *post‐hoc* PAS staining showed that the basal membrane remained intact up to day 8. The detached epithelial surface demonstrated a jagged basal surface, potentially representing detachment points from the basal membrane (*Fig*. 5). *Post‐hoc* CKAE1/AE3 staining demonstrated a total absence of keratinocytes along the unhealed wound basal membrane. The mechanical trauma of histological processing did not cause more frequent avulsion of the neo‐epithelium in healed wound surfaces from wounds exposed to TXA compared with controls.

## Discussion

Topical TXA is gaining popularity as a simple, low‐cost intervention to reduce surgical bleeding^23^. The present study has shown that that limited topical exposure to TXA is well tolerated *in vitro*. Chronic exposure may, however, exert dose‐dependent cytotoxicity, affecting wound re‐epithelialization and possibly inducing cell detachment at high concentrations.

Threshold levels found in experimental studies can act as a guide when determining a potentially safe dose and mode of topical administration *in vivo*. This study investigated a wide range of clinically relevant concentrations for topical use in various models as close to *in vivo* conditions as possible. The findings therefore add incremental evidence regarding the topical use of TXA.

There are few studies investigating possible topical toxicity from TXA. Existing publications mainly investigate chondrocyte toxicity, as topical TXA has so far been most extensively used in arthroplasties. Observed cytotoxicity is most likely a product of concentration and exposure time. Studies on the effect of topical TXA on chondrocytes and cartilage tissue suggest a threshold for toxicity of around 25 mg/ml with at least 3 h of exposure, and cells embedded in a natural (cartilage) or artificial (hydrogel) matrix are more resilient^13^, ^20^, ^24^. Furst and colleagues^25^ reported 50 per cent viability in human fibroblasts exposed to TXA 100 mg/ml and 65 per cent viability for 50 mg/ml after 100 min of exposure; this is an intermediate exposure time compared with the present 10 min for limited exposure and 24–72 h for chronic exposure. Marmotti and co‐workers^19^ found that chondrocytes, tenocytes and synoviocytes were not affected by a 2‐week exposure to TXA 7 mg/ml, whereas Bergenholtz *et al*.^26^ found that TXA concentrations of 12 mg/ml or less did not affect *in vitro* wound healing of incisional wounds through palatal mucosa from cats. This is in accordance with the present wound biopsies healing during an 8‐day exposure to TXA 6·25 mg/ml.

As prolonged exposure to TXA 25 mg/ml prevented re‐epithelialization *ex vivo*, the authors propose that the threshold level of 25 mg/ml suggested in chondrocyte studies should be lowered. Chronic exposure to 5–10 mg/ml seems to be well tolerated by chondrocytes, keratinocytes and fibroblasts, but studies are few and investigations on other tissues are largely lacking. *In vivo* studies are particularly needed.

The limited exposure resulting from the present moistening technique has not been investigated in previous studies, and these results suggest that cells are able to withstand exposure to high concentrations of TXA for a short period of time. Although limited exposure even to 100 mg/ml was well tolerated, there was a statistically non‐significant trend towards reduced viability in the cell studies, and lower healing scores in the wound model after limited exposure to 100 mg/ml. A dilution to 50 or 25 mg/ml ensures low risk of cytotoxicity. The lowest effective dose in a moistening technique needs to be explored, but 25 mg/ml has been found effective^21^.

Complete re‐epithelialization in the present human skin model was observed after limited exposure to 100 mg/ml and chronic exposure to 6·25 mg/ml, and the neo‐epithelium was no more fragile during histological processing than control neo‐epithelium. However, the total lack of wound re‐epithelialization following chronic exposure to 25 mg/ml and above in the human skin wound model could not readily be foreseen by the cytotoxicity observed in the cell studies. Thus, the present study demonstrates the importance of testing toxicity in various models, preferably as close to *in vivo* conditions as possible. Although chronic TXA exposure to 25 mg/ml caused at least a 50 per cent reduction in viability in both fibroblasts and keratinocytes by day 3 in the cell studies, both epithelium and dermis in this skin model showed no apparent histological signs of cell death (HES staining) even after 8 days of chronic exposure to 100 mg/ml. It was therefore surprising that TXA seemed to be more detrimental to an *ex vivo* re‐epithelialization process than to *in vitro* single‐cell layers. As the basal membrane was intact and the epidermal cells remained viable, TXA seemed to trigger a detachment and possibly prevented migration of keratinocytes.

Findings from other studies may suggest an antiadhesive effect of TXA. Cox and colleagues^27^ have demonstrated that the presence of high‐dose TXA both prevents fibroblast adherence and causes fibroblast detachment *in vitro*, proposing a non‐cytotoxic mechanism with changes in integrin interaction. This could also be a proposed mechanism for the present observed epithelial detachment at high concentrations. Commercial fibrin sealants supplemented with TXA have demonstrated inferior adhesive strength compared with sealants supplemented with aprotinin^25^, and have caused fewer unwanted intra‐abdominal adhesions compared with sealants without TXA in animal studies^28^, ^29^. Plasminogen can interact with integrins as an adhesive ligand, and TXA may prevent such interaction through its binding to plasminogen^30^; alternatively, there may be some unrecognized direct effect of TXA on cell attachment or migration.

If prolonged exposure to above‐threshold concentrations of TXA may be cytotoxic, prevent re‐epithelialization, reduce cell adhesions or cause cell detachment, questions are raised regarding possible reduced tensile strength in various healing tissues, delayed re‐epithelialization in large wound surfaces such as burns and split‐thickness skin grafts, and whether exposure to TXA may prevent unwanted postsurgical adhesions or capsular contractures. More research is needed to clarify these issues.

This study has several weaknesses that should be addressed. The models of topical exposure do not necessarily mimic real‐life settings. The TXA concentration after topical bolus administration will be lowered gradually due to dilution by tissue fluids and absorption. Thus, chronic exposure to an assumed constant concentration for 72 h in the cell model and for 8 days in the human skin wound model represents a much more intensive exposure than would ever be the case *in vivo*. Similarly, limited exposure for only 10 min may be less than what might be the case after moistening a wound surface. However, when discarding the TXA fluid without rinsing, a small amount of TXA will inevitably remain in the wells. It was found that, after adding fresh growth medium, both cells and wound models were chronically exposed to a concentration that was 6–10 per cent of the original. This remnant was not further diluted, as it would have been in an *in vivo* setting. Thus the limited exposure involved a low‐dose chronic exposure. This method was found preferable to rinsing, owing to the possibility of underestimating an exposure and inflicting mechanical stress.

Keratinocytes and fibroblasts have different growth patterns. Keratinocytes are particularly fragile and more challenging to grow in cell culture^31^. The results from the MTT and LDH assays are proportional to the number of cells, and therefore varied between series. This may have contributed to large standard deviations in the final results.

According to the authors' experience, the CelluTome™ epidermal harvesting system for the human skin wound model creates standardized superficial wounds, but this needs confirmation in other studies. The present novel healing score must also be confirmed. Healing may be affected by the individual quality of the donated skin, and the score sheet is a compilation of biopsies from various patients. Standardized models for deeper wounds are also needed to evaluate the process of granulation and fibroblast activity.

This study has investigated the effect of both prolonged and limited exposure of a wide range of clinically relevant TXA concentrations in human keratinocytes and fibroblasts using assays measuring both viability and cytotoxicity, as well as in an *ex vivo* human skin wound model with standardized superficial wounds. This wide approach is a significant strength. Based on the present findings, the authors propose that the planned mode of administration should direct the TXA concentration. They recommend that bolus administrations of topical TXA should not exceed a concentration of 5–10 mg/ml, and propose a TXA concentration of 25–50 mg/ml when moistening a surgical wound. More research is needed to determine the lowest effective concentrations of topical TXA.

## Supporting information


**Appendix S1** Details of cell culture viability and cytotoxicity assays
**Appendix S2** Histology staining protocols
**Table S1** Underlying data for *Fig*. 3 *a–d*
**Table S2** Underlying data for *Fig*. 3 *e–h*
**Table S3** Cell studies, limited exposure to tranexamic acid from powder dissolved in medium
**Table S4** Underlying data for *Fig*. 4
