# Supplementary material for: Cytotoxicity and effect on wound re‐epithelialization after topical administration of tranexamic acid
Source: BJS Open. 2019 Sep 26;3(6):840–51. doi: 10.1002/bjs5.50192 (PMC6887721; doi:10.1002/bjs5.50192)
Supplement: Supplementary file 1 — Appendix S1 Details of cell culture viability and cytotoxicity assays Appendix S2 Histology staining protocols Table S1 Underlying data for Fig. 3 a–d Table S2 Underlying data for Fig. 3 e–h Table S3 Cell studies, limited exposure to tranexamic acid from powder dissolved in medium Table S4 Underlying data for Fig. 4 [file BJS5-3-840-s001.docx]

**BJS5_50192**

**Cytotoxicity and effect on wound re-epithelialization after topical administration of tranexamic acid**

**T. A. Eikebrokk^1^, B. S. Vassmyr^1^, K. Ausen^2,4^, C. Gravastrand^3^, O. Spigset^3,5^ and B. Pukstad**

#

# Appendix S1 Details of cell culture viability and cytotoxicity assays

Adult Human Epidermal Keratinocytes (catalogue no. C0055C, Gibco™, Carlsbad, CA, USA) and adult Human Dermal Fibroblasts (catalogue no. C0135C, Gibco™) were stored in liquid nitrogen and upon use placed in a 37°C water bath for thawing. The cell suspensions were transferred to two 75 cm^2^ culture flasks with 15 ml growth medium which had been preheated to 37°C. The keratinocytes were cultured in EpiLife™ medium supplemented with Human Keratinocyte Growth Supplement (HKGS) (Gibco™), 10 μg/ml gentamicin and 0.25 μg/ml amphotericin B (Gibco™). The fibroblasts were cultured in Dulbecco modified Eagle´s medium (DMEM, Lonza, Walkersville, MD, USA) with 10 % fetal calf serum (FCS), 700 µM L-glutamine (Sigma Aldrich St. Louis, MO, USA), 10 μg/ml gentamicin and 0,25 μg/ml amphotericin B. The culture flasks were incubated in a humidified 37°C, 5 % CO_2_ incubator. After 24 hours, the old medium was discarded and fresh medium added to the flasks. Cell proliferation was monitored by studying the cell confluence in an inverted light microscope. After reaching 70-90 % cell confluence, the growth medium was removed and the cells were washed with 10 ml phosphate buffered saline (PBS). 3 ml of 0, 25 % trypsin/EDTA was added and the flasks were incubated at room temperature, and regularly checked using an inverted light microscope. When most of the cells were rounded up, the flasks were given a tap on the side and 15 ml fresh preheated fibroblast medium with 10 % FCS was added to inactivate the trypsin. The cell suspensions were transferred to two 50 ml centrifuge tubes and centrifuged for 5 minutes, 470 x g at room temperature. The supernatant was discarded and the cell pellets were re-suspended in 5 ml fresh growth medium. *Countess*™ *Automated Cell Counter* (Invitrogen^TM^, Fisher Scientific, Oslo, Norway) was used to count the cells, and fresh growth medium was added to dilute the cells to a concentration of 5x10^4^ cells/ml. The cells were seeded in 96 well plates (Corning®, Sigma Aldrich) in a concentration of 5x10^3^ cells/well. When the seeded cells reached a confluence of around 80%, the old growth medium was discarded. From this point, low condition growth medium was used. For the keratinocytes, this implies the removal of HKGS, while for the fibroblasts FCS was reduced to 1%. This was done to avoid interference of serum with the LDH-assay.

## *Cytotoxicity assays*

### **Lactate dehydrogenase (LDH)**

50 μl supernatant was transferred to a 96 well plate. A low control of pure low condition medium was added, as well as a high control of supernatant from cells lysed with Triton X (1% in medium). 50 μl of LDH reaction mixture (Cytotoxicity Detection Kit (LDH), Roche) was added to each well. The plates were incubated in the dark for 30 min at room temperature before 25 μl of 1M HCl was added to each well. The absorbance was measured at 490 and 655 nm, using *POLARstar Omega* (BMG LABTECH, Ortenberg, Germany)*.*

### **3-(4,5-Dimethylthiazol-2-yl)-2,5-Diphenyltetrazolium Bromide (MTT)**

The metabolic activity assay MTT was chosen for viability measurements. MTT was diluted to 10% in fresh low condition medium. 100 μl of the MTT solution was added to each well. After incubation at 37°C for three hours, the medium was removed and the formazan crystals were solubilized by addition of 50 μl isopropanol with HCl per well. The absorbance was measured at 450 and 570 nm, using *VICTOR™, Multilabel reader* (PerkinElmer, Inc., Waltham, MA, USA)*.*

### **Calculation of viability and cytotoxicity**

An average value for each concentration was calculated. Background absorbance was subtracted. In the MTT assay, the average optical density (OD) value from cells exposed to medium only (medium control) was set to 100% cell viability for both acute and chronic TXA exposure. Cell viability was calculated by comparing OD values from cells exposed to different TXA concentrations (sample) to medium control (Formula 1). In the LDH assay, the average OD value from cells lysed with Triton-X (high control) was set to 100% cytotoxicity. Cytotoxicity was then calculated according to Formula 2.

$$\mathbf{Formula 1}: \% viability= \frac{sample}{medium control} x 100\%$$

$$\mathbf{Formula 2}\boldsymbol{:} \% cytotoxicity=\frac{sample-medium control}{high control-medium control} x 100\%$$

**Appendix S2 Histology staining protocols**

**HES**

**4 μm thin formalin fixed, paraffin embedded tissue sections**

Equipment and reagents

-Automated slide stainer: Tissue - Tek © Prisma™ **(**Sakura Finetek, Alpen aan den Rijn, the Netherlands)

-Counterstaining: Hematoxylin RHD-1475-100 (CellPath/ Chemi-Teknik AS, Oslo, Norway)

-Erythrosine 239 (RAL diagnostics/ VWR, Oslo, Norway)

-Saffron (VWR, Oslo, Norway)

-Mounting medium: Glas Tissue Mount Medium (Sakura Finetek, Alpen aan den Rijn, the Netherlands)

1. Deparaffinize sections in Tissue Clear x 3, each 3 minutes.

2. Rehydrate in graded ethanol to water, (ethanol 100% x 3, ethanol 96% and finally 80% to water) each 1 minute.

3. Wash in water, 1 minute.

4. Stain in Haematoxylin, 5 minutes.

5. Rinse in running water, 8 minutes.

6. Stain in Erythrosin, 5 minutes.

7. Rinse in running water, 1 minute

8. Dehydrate in graded ethanol (80%, 96% - 100% ethanol) each 1 minute.

9. Stain in Saffron, 5 minutes.

10. Rinse in ethanol 100 % x 3, each 1 minute.

11. Tissue Clear x 3, each 1 min.

12. Dispense mounting medium and coverslip

**PAS**

**4 μm thin formalin fixed, paraffin embedded tissue sections**

Equipment and reagents

-Automated slide stainer: Tissue - Tek © Prisma™ **(**Sakura Finetek, Alpen aan den Rijn, the Netherlands)

-1% Periodic Acid (Merck / VWR, Oslo, Norway)

-Schiff’s Reagent (Merck / VWR, Oslo, Norway)

-Haematoxylin RHD-1475-100,(CellPath/ Chemi-teknik AS, Oslo, Norway)

-Mounting medium: Tissue Mount Medium (Sakura Finetek, Alpen aan den Rijn, the Netherlands)

1. Deparaffinize sections in Tissue Clear x 3, each 3 minutes.

2. Rehydrate in graded ethanol to water, (ethanol 100% x 3, ethanol 96% and finally 80% to water) each 1 minute.

3. Treat with 1% Periodic Acid for 15 minutes

4. Rinse well in distilled water

5. Cover with Schiff’s Reagent at room temperature for 10 minutes

6. Rinse in lukewarm running tap water for 5 minutes

7. Stain in Hematoxylin, 4 minutes

8. Rinse in lukewarm running tap water for 5 minutes

9. Dehydrate in graded ethanol (80%, 96% - 100% ethanol) each 1 minute.

10. Tissue Clear x 3, each 1minute.

11. Dispense mounting medium and coverslip

**Immunohistochemical staining for CKAE1/AE3**

**4 μm thin formalin fixed, paraffin embedded tissue sections**

Equipment and reagents (Dako, Agilent Technologies, Glostrup, Denmark)

-Autostainer Plus, Dako

-PT-link, Dako

-Target Retrieval Solution (TRS, pH9), Dako K8004

-Washbuffer: TBS (Tris Buffer Saline), Dako K8007

-Peroxidase block: H2O2, Dako S2023

-Antibody diluent: Dako S2022

-Primary antibody: Cytokeratin, clone AE1/AE3 (CKAE1/AE3), Dako M3515

-Detection system: Peroxidase labelled polymer with DAB substrate chromogen: EnVision+System- HRP (DAB) for use with Mouse primary antibodies, Dako K4007

1. Place sections on Superfrost Plus glasses, dry overnight in 37*C and 1 hour in 60*C.

2. Deparaffinize sections in Tissue Clear x 3, each 3 minutes.

3. Rehydrate in graded ethanol to water, (ethanol 100% x 3, ethanol 96% and finally 80% to water) each 1 minute.

4. Immerse in TRS and pretreat for 20 minutes at 97*C.

5. Rinse in TBS buffer

6. Treat with H2O2 for 10 minutes

7. Rinse in TBS buffer

8. Incubate for 30 minutes in diluted primary antibody CKAE1/AE3 1:50

9. Rinse in TBS buffer

10. Incubate for 30 minutes in secondary antibody EnVision+MouseHPR

11. Rinse in TSB buffer x 2

12. Incubate for 2 x 5 minutes in chromogen solution (DAB)

13. Rinse in deionized water x 2

14. Counterstain with hematoxylin for 1 minute

15. Rinse in water for 5 minutes

16..Dehydrate in graded ethanol (80%, 96% - 100% ethanol) each 1 minute.

17. Tissue Clear x 3, each 1 minute.

18. Dispense mounting medium and coverslip

**Table S1** Underlying data for *Fig. 3a–d*

|  | | **TXA concentration** | | | | |
| --- | --- | --- | --- | --- | --- | --- |
|  | | **100 mg/ml** | **50 mg/ml** | **25 mg/ml** | **12.5 mg/ml** | **6.25 mg/ml** |
| Keratinocytes MTT  viability % | Day 1 | -93.8 ± 9.8 p=0.001 | -90.8 ± 8.9 p<0.001 | -55.0 ± 8.5 p<0.001 | -38.2 ± 11.1 p=0.015 | -24.8 ± 10.4 p=0.018 |
|  | Day 2 | -99.1 ± 16.8 p<0.001 | -100.6±15.9 p<0.001 | -62.6 ± 16.6 p=0.007 | -38.5 ± 20.3 p=0.070 | -25.1 ± 24.9 p=0.12 |
|  | Day 3 | -94.4 ± 25.8 p<0.001 | -95.8 ± 25.9 p<0.001 | -68.3 ± 25.0 p=0.010 | -53.7± 15.3 p<0.001 | -38.5 ± 13.8 p<0.001 |
|  |  |  |  |  |  |  |
| Fibroblasts MTT  viability % | Day 1 | -82.5 ± 13.8 p=0.005 | -57.6 ± 11.3  p<0.001 | -43.9 ± 12.4  p<0.001 | -20.9 ± 14.3  p<0.001 | -19.9 ± 12.0  p=0.036 |
|  | Day 2 | -90.9 ± 20.2 p=0.019 | -88.2 ± 6.8 p<0.001 | -46.5 ± 12.2  p=0.026 | -31.2 ± 16.4  p=0.081 | -21.9 ± 16.0  p=0.14 |
|  | Day 3 | -101.0±10.6 p=0.001 | -99.2 ± 14.4  p=0.004 | -45.1 ± 18.7  p=0.053 | -33.1 ± 16.5 p=0.051 | -28.1 ± 15.9  p=0.087 |
|  |  |  |  |  |  |  |
| Keratinocytes LDH  cytotoxicity % | Day 1 | 82.4 ± 10.6  p=0.005 | 57.5 ± 12.5  P=0.016 | 16.5 ± 5.4  p=0.028 | 5.6 ± 4.2 p=0.111 | 1.4 ± 1.9 p=0.011 |
|  | Day 2 | 112.5 ± 15.9  p=0.003 | 125.3 ± 19.3  p=0.007 | 45.4 ± 11.2  p=0.017 | 11.6 ± 10.2 p=0.130 | 4.8 ± 8.0 p=0.212 |
|  | Day 3 | 71.3 ± 28.6  p=0.042 | 102.3±39.7  p<0.001 | 41.3 ± 13.3 p=0.019 | 18.9 ± 9.8 p=0.037 | 7.2 ± 9.2 p=0.008 |
|  |  |  |  |  |  |  |
| Fibroblasts LDH  cytotoxicity % | Day 1 | 42.9 ± 13.9 p=0.033 | 2.1 ± 3.5 p=0.31 | 0.7 ± 3.9  p=0.8 | -1.2 ± 1.2 p=0.11 | -1.2 ± 1.1 p=0.001 |
|  | Day 2 | 56.7 ± 12.7  p=0.016 | 37,5 ± 11.2 p=0.036 | 5.4 ± 3.6 p=0.086 | 0.9 ± 2.5 p=0.56 | 0.3 ± 1.8 p=0.78 |
|  | Day 3 | 84.4 ± 13.4  p=0.011 | 68.1 ± 13.2 p=0.014 | 7.0 ± 2.3 p<0.001 | 2.6 ± 2.6 p=0.087 | 1.9 ± 2.6 p=0.277 |

Chronic exposure to TXA powder dissolved in medium. Mean difference ± SD for both cytotoxicity and viability between each concentration of TXA and the corresponding control value. Significance p calculated using a linear mixed model with series as random effect. Only differences >10% were considered clinically significant and marked with an asterisk in Figure 3.

**Table S2** Underlying data for *Fig. 3e–h*

|  | | **TXA concentration** | | | | |
| --- | --- | --- | --- | --- | --- | --- |
|  | | **100 mg/ml** | **50 mg/ml** | **25 mg/ml** | **12.5 mg/ml** | **6.25 mg/ml** |
| Keratinocytes MTT  viability % | Day 1 | -11.7 ± 34.7 p=0.491 | 10.3 ± 38.4 p=0.595 | 20.9 ± 36.3 p=0.299 | 26.0 ± 32.7 p=0.164 | 29.1 ± 32.2 p=0.165 |
|  | Day 2 | -23.1 ± 20.9 p=0.046 | -8.5 ± 23.7 p=0.423 | 6.1 ± 24.3 p=0.622 | 3.0 ± 17.9 p=0.649 | 6.4 ± 20.0 p=0.469 |
|  | Day 3 | -29.0 ± 26.4 p=0.094 | 1.9 ± 25.1 p=0.777 | 9.6 ± 20.2 p=0.048 | 13.6 ± 22.8 p=0.015 | 10.9 ± 16.5 p=0.008 |
|  |  |  |  |  |  |  |
| Fibroblasts MTT  viability % | Day 1 | -25.6 ± 14.2 p=0.083 | -14.6 ± 13.2 p=0.105 | -6.4 ± 8.8 p=0.014 | -3.2 ± 7.5 p=0.114 | 0.5 ± 6.7 p=0.759 |
|  | Day 2 | -22.6 ± 27.3 p=0.331 | -12.6 ± 27.7 p=0.537 | -10.4 ± 18.7 p=0.391 | -3.2 ± 9.4 p=0.202 | 0.5 ± 7.2 p=0.776 |
|  | Day 3 | -28.4 ± 28.7 p=0.263 | -12.4 ± 25.3 p=0.523 | -4.5 ± 18.3 p=0.717 | 1.1 ± 19.2 p=0.932 | -1.2 ± 18.1 p=0.919 |
|  |  |  |  |  |  |  |
| Keratinocytes LDH  cytotoxicity % | Day 1 | -1.6 ± 5.9 p=0.620 | -3.6 ± 4.9 p=0.134 | -2.9 ± 3.3 p=0.100 | -3.3 ± 3.1 p=0.121 | -2.7 ± 5.2 p=0.286 |
|  | Day 2 | 3.5 ± 7.1 p=0.382 | 1.0 ± 5.8 p=0.685 | 0.7 ± 4.5 p=0.634 | 1.9 ± 5.8 p=0.385 | 2.8 ± 7.0 p=0.432 |
|  | Day 3 | 9.9 ± 11.9 p=0.064 | 2.3 ± 7.8 p=0.313 | 2.4 ± 6.3 p=0.206 | 1.9 ± 6.0 p=0.340 | 0.3 ± 4.9 p=0.895 |
|  |  |  |  |  |  |  |
| Fibroblasts LDH  cytotoxicity % | Day 1 | -0.9 ± 1.2 p=0.183 | -1.8 ± 0.8 p=0.038 | -1.6 ± 0.8 p=0.054 | -1.8 ± 1.5 p=0.093 | -1.5 ± 1.1 p=0.056 |
|  | Day 2 | 0.3 ± 1,9 p=0.825 | -1.0 ± 0.9 p=0.080 | -0.6 ± 0.9 p=0.253 | -0.8 ± 1.0 p=0.167 | -0.2 ± 0.8 p=0.255 |
|  | Day 3 | 5.6 ± 1.8 p=0.027 | -0.5 ± 1.9 p=0.642 | -0.8 ± 1.3 p=0.034 | -1.1 ± 1.7 p=0.155 | -1.2 ± 1.2 p=0.080 |

Limited exposure to TXA vials (100 mg/ml TXA in sterile water) diluted in 0.9% NaCl. Mean difference ± SD for both cytotoxicity and viability between each concentration of TXA and the corresponding control value. Significance p calculated using a linear mixed model with series as random effect. Only differences >10% were considered clinically significant and marked with an asterisk in Figure 3.

**Table S3** Cell studies, limited exposure to tranexamic acid from powder dissolved in medium

|  | | **TXA concentration** | | |
| --- | --- | --- | --- | --- |
|  | | **100 mg/ml** | **50 mg/ml** | **25 mg/ml** |
| Keratinocytes MTT  viability % | Day 1 | -44.43 ± 21.46 p=0.063 | -30.47 ± 16.05 p=0.074 | -17.95 ± 16.41 p=0.164 |
|  | Day 2 | -27.50 ± 17.58 p=0.112 | -14.39 ± 17.07 p=0.265 | -2.74 ± 16.91 p=0.785 |
|  | Day 3 | -19.72 ± 25.06 p=0.212 | 0.26 ± 21.07 p=0.978 | -3.83 ± 13.58 p=0.293 |
|  |  |  |  |  |
| Fibroblasts MTT  viability % | Day 1 | -5.11 ± 15.14 p=0.345 | 2.86 ± 9.67  p=0.592 | 6.12 ± 8.16  p=0.011 |
|  | Day 2 | -24.09 ± 12.45 p<0.001 | -3.81 ± 11.90 p=0.545 | -2.86 ± 6.47  p=0.336 |
|  | Day 3 | -22.82 ± 12.86 p=0.098 | -4.95 ± 14.44  p=0.634 | --5.37 ± 9.55  p=0.443 |
|  |  |  |  |  |
| Keratinocytes LDH  cytotoxicity % | Day 1 | 0.36 ± 1.52  p=0.369 | -0.17 ± 1.13  p=0.808 | -0.93 ± 0.58  p=0.031 |
|  | Day 2 | 2.58 ± 5.34  p=0.540 | 0.91 ± 5.48  p=0.825 | 1.60 ± 5.60  p=0.708 |
|  | Day 3 | 4.83 ± 9.03  p=0.342 | -1.65 ± 9.20  p<0.613 | -4.20 ± 7.01 p=0.036 |
|  |  |  |  |  |
| Fibroblasts LDH  cytotoxicity % | Day 1 | 2.14 ± 2.29  p=0.230 | 0.31 ± 0.90 p=0.323 | 0.08 ± 0.14 p=0.040 |
|  | Day 2 | 2.21 ± 2.36  p=0.163 | 0.89 ± 1.41 p=0.028 | 0.16 ± 0.32 p=0.248 |
|  | Day 3 | 1.73 ± 2.34 p=0.209 | 0.51 ± 2.23 p=0.636 | -0.50 ± 1.32 p=0.942 |

Mean difference ± SD for both cytotoxicity and viability between each concentration of TXA and the corresponding control value. Significance p calculated using a linear mixed model with series as random effect.

**Table S4** Underlying data for *Fig. 4*

|  | | **TXA concentration** | | | | | | |
| --- | --- | --- | --- | --- | --- | --- | --- | --- |
|  | | **Chronic exposure** | | | |  | **Limited exposure** | |
|  | | **Medium**  **control** | **100 mg/ml** | **25 mg/ml** | **6.25 mg/ml** |  | **100 mg/ml** | **NaCl 0.9%**  **control** |
| **Healing score** | |  |  |  |  |  |  |  |
| Mean ± SD  (range)  n=number of samples  p=significance compared to respective control | Day 1 | 1.88 ± 0.99  (1.0-3.0)  n=8 | 0.00 ± 0.00 (0.0-0.0)  n=9  p=0.010 | 0.07 ± 0.19 (0.0-0.5)  n=7  p=0.016 | 0.28 ± 0.44  (0.00-1.00) n=9  p=0.027 |  | 0.00 ± 0.00 (0.0-0.0)  n=6  p=0.317 | 0.3 ± 0.45 (0-0-1.0)  n=5 |
|  |  |  |  |  |  |  |  |  |
|  | Day 4 | 4.8 ± 1.25  (3.0–6.0)  n=8 | 0.00 ± 0.00 (0.0-0.0)  n=9  p=0.010 | 0.22 ± 0.44 (0.0-1.0)  n=9  p=0.011 | 2.75 ± 0.76 (2.0-4.0)  n=6  p=0.042 |  | 4.11 ± 1.53 (2.0-6.0)  n=9  p=0.915 | 4.07 ± 0.45 (3.5–5.0)  n=7 |
|  |  |  |  |  |  |  |  |  |
|  | Day 8 | 5.78 ± 0.44 (5.0-6.0)  n=9 | 0.00 ± 0.00 (0.0-0.0)  n=9  p=0.005 | 0.00 ± 0.00 (0.0-0.0)  n=9  p=0.005 | 3.78 ± 1.06 (2.5–5.0)  n=9  p=0.011 |  | 3.43 ± 1.13 (1-4)  n=7  p=0.066 | 4.60 ± 1.14 (3.0-6.0)  n=5 |

Healing scores in the human skin wound model according to the scoring system in Figure 2. Chronic exposure to TXA from powder dissolved in medium with medium control, and limited exposure from vial TXA 100 mg/ml in sterile water with NaCl 0.9% as control. Mean healing score is compared to its corresponding control value using a Wilcoxon signed rank test.
